# Effectiveness and safety of Yufengningxin for treating coronary heart disease angina: A protocol for a systematic review and meta-analysis

**DOI:** 10.1097/MD.0000000000023911

**Published:** 2020-12-24

**Authors:** Chao Liu, Jing Bai, Lanchun Liu, Jialiang Gao, Jie Wang

**Affiliations:** a Guang’anmen Hospital, China academy of Chinese medical Sciences; b Beijing University of Chinese Medicine, Beijing; c Shaanxi University of Chinese Medicine, Shaanxi, China.

**Keywords:** coronary heart disease angina, meta-analysis, protocol, systematic review, Yufengningxin

## Abstract

**Background:**

More than 11 million people suffer from coronary heart disease (CHD) angina in China, showing high morbidity and mortality rates. Yufengingxin (YFNX) is a commonly used Chinese patent medicine in CHD angina treatment. The purpose of this protocol is to evaluate the effectiveness and safety of YFNX for the treatment of CHD angina.

**Methods:**

A systematic search of randomized controlled trials related to the effectiveness and safety of YFNX in the treatment of CHD angina will be performed from relevant databases, including PubMed, Cochrane Library, EMBASE, Chinese National Knowledge Infrastructure (CNKI), Wanfang Database, Chinese Scientific Journal Database (VIP) and Chinese Biomedical Literature Database (CBM). We will screen all the literatures from the database inception to November 1, 2020. The data including study ID, study characteristics, methodological information, patients information, interventions, comparisons and outcomes will be extracted. The frequency and duration of angina attacks will be served as the primary outcome. Review Manager 5.3 and STATA 14.0 software will be used for data analysis.

**Conclusion:**

This systematic review will provide strong evidence for the effectiveness and safety of YFNX in the treatment of CHD angina.

**Trial registration number:**

INPLASY2020110040.

## 1 Introduction

Coronary heart disease (CHD) angina, a common cardiovascular disease, is caused by coronary artery stenosis or spasm, which leads to myocardial ischemia and hypoxia.^[1]^ The characteristics of angina pectoris is recurred deep chest pain, which could radiate to the patients shoulder, neck, or inner arm. Since the end of the last century, cardiovascular disease has become a leading cause of death among Chinese residents.^[2]^ At present, 11396,000 people in China suffer from CHD. The mortality rate of urban/rural residents reaches 115.32 per 100,000 and 122.04 per 100,000, respectively, and it shows an increasing trend year by year. Every year, nearly 910,000 patients receive percutaneous coronary intervention therapy.^[3]^ CHD seriously affects peoples quality of life and puts a huge economic burden on society.^[4]^

Nitrates are the most commonly used drugs for relieving angina pectoris. However, the use of nitrates has been found to have some side effects in clinical practice, such as dizziness, blushing, nausea, and loss of appetite.^[5]^ Therefore, to explore novel strategies for alleviating symptoms of angina, especially those with no obvious side effects, becomes an urgent task. Recently, a high-quality systematic review concluded that traditional Chinese medicine in the treatment of CHD might be effective in alleviating angina symptoms, abnormal myocardial perfusion and neurological deficits, and could be used as a complementary therapy for the treatment of CHD.^[6]^

Yufengningxin (YFNX), which is extracted and processed from Radix Puerariae, is a commonly used Chinese patent medicine for the treatment of CHD. It has the functions of relieving spasm and relieving pain, enhancing brain and coronary blood flow, and improving angina symptoms. Because of its remarkable effects, it was included in the Pharmacopoeia of the People's Republic of China in the 2020 edition.^[7]^ High-performance liquid chromatography analysis showed that the main active ingredient of YFNX was puerarin, which should not be less than 13 mg/tablet as stipulated by the Pharmacopoeia.^[8,9]^ Puerarin (7, 4-dihydroxyisoflavone-8b-glucopyranoside) is a kind of isoflavone compound, which has a strong activity in the cardiovascular system. It can dilate the coronary blood vessels, reduce the oxygen consumption of the myocardium, inhibit the inflammatory response, and protect the cardiomyocytes.^[10–12]^

Some studies have confirmed that YFNX can significantly reduce the frequency and duration of angina in patients with CHD, and improve clinical efficacy.^[13,14]^ At the same time, YFNX can also reduce the risk factors of CHD, including lowering blood pressure and low-density lipoprotein cholesterol.^[15,16]^ In addition, animal experiments have further confirmed that YFNX has a protective effect on myocardial ischemia induced by isoproterenol hydrochloride in mice, improving the hypoxia tolerance of mice and prolonging the average survival time of mice with myocardial hypoxia.^[17]^ However, there is still no strong evidence to indicate the effectiveness and safety of YFNX in treating CHD angina. Therefore, a high quality systematic review and meta-analysis of the effectiveness and safety of YFNX in treating CHD angina will be carried out to provide a reference for clinical use.

## 2 Methods

This review will be performed and reported in strict accordance with the Preferred Reporting Items for Systematic Reviews and Meta-Analysis (PRISMA) Statement.^[18]^ The current protocol was registered with INPLASY (registration number: INPLASY2020110040). If there are any adjustments in this study, we intend to make revisions and updates in the final publication.

### 2.1 Eligible criteria for study selection

#### 2.1.1 Types of studies

We will include all relevant randomized controlled trials (RCTs) regarding the application of YFNX to the treatment of CHD angina, with no limitation of the language and the type of publication.

#### 2.1.2 Types of participants

By following the World Health Organization (WHO) diagnostic criteria for CHD, 2 kinds of CHD angina will be included, including stable angina pectoris (SAP) and unstable angina (UA). We will not consider the patients gender, age, ethnicity and region.

#### 2.1.3 Types of interventions

The control group accepted conventional therapy, such as aspirin, clopidogrel, β-blockers, statins, calcium channel blockers, renin-angiotensin-aldosterone system blockers, nitrates, etc. And the intervention group took YFNX combined with conventional therapy. The various dosage forms of YFNX will be included, such as tablets, capsules, dripping pills, granules and so on. Its dosage and duration will not be limited. The study in which other Chinese medicines were used will be excluded.

#### 2.1.4 Types of outcome measures

##### 2.1.4.1 Primary outcomes

The primary outcomes will be the frequency and duration of angina attacks.

##### 2.1.4.2 Secondary outcomes

The secondary outcomes will include clinical efficacy rate, the efficacy rate in an electrocardiogram, scores of TCM syndrome, blood pressure, low-density lipoprotein cholesterol, fibrinogen, haematocrit and adverse events.

### 2.2 Search methods for the identification of studies

#### 2.2.1 Electronic searches

We will perform a systematic search of the relevant databases, including PubMed, Cochrane Library, EMBASE, Chinese National Knowledge Infrastructure (CNKI), Wanfang Database, Chinese Scientific Journal Database (VIP) and Chinese Biomedical Literature Database (CBM), from their inception to November 1, 2020. The English search terms include YFNX, CHD, angina pectoris and RCTs. The detailed search strategy for PubMed is shown in Table 1, and similar terms will be used in the other databases.

**Table 1:** Search strategy for PubMed.

#1	Coronary heart disease [Title/Abstract]
#2	Coronary artery disease [Title/Abstract]
#3	CHD [Title/Abstract]
#4	Angina [Title/Abstract]
#5	Angina pectoris [Title/Abstract]
#6	Stable angina pectoris [Title/Abstract]
#7	Unstable angina [Title/Abstract]
#8	#1 or #2 or #3 or #4 or #5 or #6 or #7
#9	yufengninxin [Title/Abstract]
#10	YFNX [Title/Abstract]
#11	#9 or #10
#12	randomized controlled trial [All Fields]
#13	controlled clinical trial [All Fields]
#14	randomized [All Fields]
#15	#12 or #13 or #14
#16	#8 and #11 and #15

#### 2.2.2 Search for other resources

We will also retrieve grey documents and clinical trial registers, such as WHO International Clinical Trials Registry Platform (ICTRP), the Chinese Clinical Trial Register (Chi CTR) and the Clinical Trials, to supplement the electronic databases.

### 2.3 Data collection and analysis

#### 2.3.1 Selection of studies

**Figure 1:**
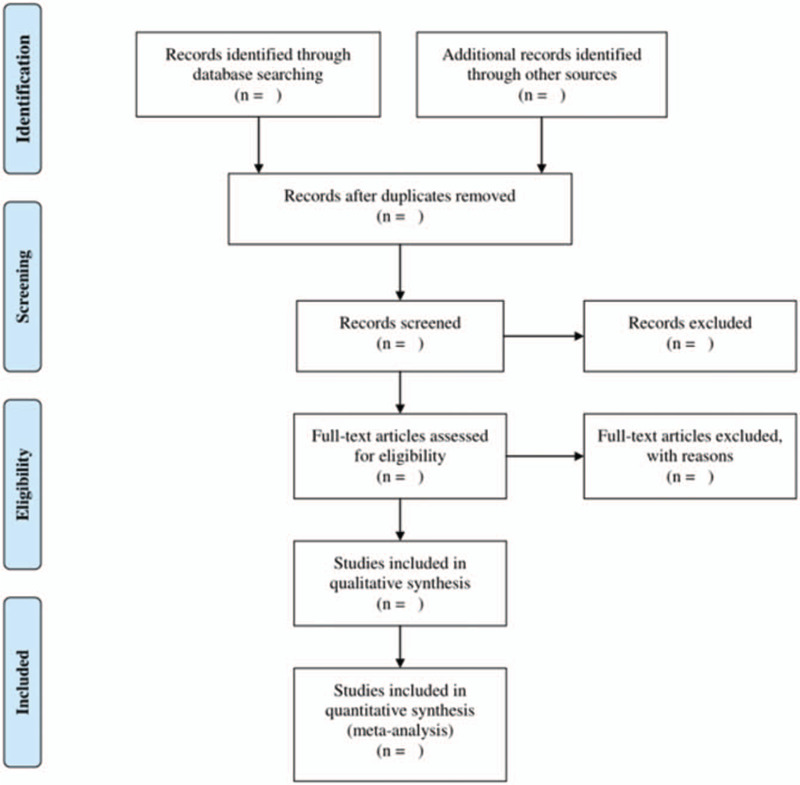
Flow chart of preferred reporting items for systematic review and meta-analysis (PRISMA).

We will import all studies into Endnote X9 software. Two researchers (Chao Liu and Jing Bai) will independently extract the potentially eligible studies by screening the titles and abstracts in accordance with the inclusion criteria. After removing irrelevant and duplicate studies, they will further scan the full text to assess their eligibility. All studies selected by the researchers will be cross-checked, and if there are any disagreements, they will be discussed and solved by a third author (Jie Wang). The literature screening and selection processes will be performed in accordance with the PRISMA flow chart shown in Figure 1.

#### 2.3.2 Data extraction and management

Two researchers (Chao Liu and Jing Bai) will independently extract and manage the data from the finally included studies according to the pre-defined data collection form. The form will include study ID, study characteristics (authors name, country, title, publication year, and so on), methodological information (randomization, allocation hiding, blinding, selective reporting, loss of follow-up and any other bias information), patients information (numbers, age, sex, diagnostic criteria, and baseline disease severity), interventions and comparisons (dosage form, dose, frequency, and duration), and outcomes (primary outcomes and secondary outcomes). If there is any unclear or missing information, the author of the original study will be consulted. Any disagreements will be solved through discussion.

#### 2.3.3 Assessment of risk of bias in included studies

The Cochrane “risk of bias” tool will be used to evaluate the risk of bias in each included study by 2 independent authors. The main items include selection bias (random sequence generation, allocation concealment), performance bias, detection bias, attrition bias, reporting bias and other biases. According to the actual situation of the research, each bias is divided into 3 levels, including low risk, high risk, and unclear. If there are any disagreements in the assessment, we will reach a consensus by discussing and negotiating with a third author (Lanchun Liu).

#### 2.3.4 Measures of treatment effect

We will use the mean difference (MD) and 95% confidence interval (CI) to analyze the continuous data. For the dichotomous data, the rate ratio (RR) and the 95% CI will be used to expressed the results.

#### 2.3.5 Dealing with missing data

As for the missing or incomplete information in the included studies, we will contact the author to obtain the missing or incomplete information by email or other means. If the information is not accessible, the impact of the missing data should be discussed, and we will only analyze the available data.

#### 2.3.6 Assessment of heterogeneity

The *I*^2^ test will be used to evaluate the statistical heterogeneity. If *I*^2^ ≤ 50%, that means there is no significant heterogeneity among studies and a fixed-effects model should be used to calculate the effect size; otherwise, it means there exists significant heterogeneity.

#### 2.3.7 Data synthesis and analysis

The RevMan 5.3 software (Cochrane Collaboration, Copenhagen, Denmark) will be used for data synthesis. If small heterogeneity (*I*^2^ ≤ 50%) exists between the studies, the fixed-effects model will be used for meta-analysis. Conversely, if evident heterogeneity is found, a random-effects model will be applied to evaluate the outcome data, and a subgroup, sensitivity analysis or descriptive analysis will be conducted to analyze the potential reasons for heterogeneity. If quantitative synthesis is not appropriate, we will perform a descriptive analysis.

#### 2.3.8 Publication bias

If there are more than 10 trials in the study, the funnel plot analysis will be performed to evaluate publication bias. In the case of fewer than 10 trials, we will conduct a quantitative analysis through the Egger test by using the STATA 14.0 software.

#### 2.3.9 Subgroup analysis

If there have obvious heterogeneity and more than 10 trials among the included studies, a subgroup analysis based on the type of angina pectoris, the dosage form of YFNX and adverse events should be conducted.

#### 2.3.10 Sensitivity analysis

To ensure the robustness of analytical conclusions, we need to perform a sensitivity analysis by eliminating each study individually or changing the category of the effect model.

#### 2.3.11 Grading the quality of evidence

The GRADE tool^[19]^ will be used to classify the quality of evidence in the systematic review into 4 levels: “high quality”, “moderate quality”, “low quality”, and “extremely low quality”.

#### 2.3.12 Ethics and dissemination

This article collects published articles for data analysis and does not involve the individual privacy of patients, so ethical approval is not necessary. The results of this review will be presented at relevant conferences and published in peer-reviewed journals.

## 3 Discussion

CHD angina affects physical function and the quality of life, and imposes a broad burden on both patients and caregivers. YFNX has significant clinical effects, and its main active ingredient puerarin can protect against myocardial ischemia/reperfusion injury by inhibiting inflammation^[10]^ and autophagy via the Akt signaling pathway,^[20]^ increasing superoxide dismutase, decreasing creatine kinase and methylene dioxyamphetamine.^[11]^ Therefore, we designed the systematic evaluation protocol by using the latest data to test the effectiveness and safety of YFNX in the treatment of CHD angina. It is hoped that this study could find more rigorous medical evidence for the application of YFNX to the treatment of CHD angina, thus providing a reference for clinical practice. However, there are still some potential limitations in this study. First, there still lacks of a high quality, multi-center and large sample clinical trial, which will affect the authenticity of the evidence. Second, different dosage forms and doses of YFNX may lead to significant heterogeneity of results. Therefore, more rigorous, large-sample, high-quality RCTs should be carried out in future studies to confirm the clinical efficacy of YFNX.

## Author contributions

**Conceptualization:** Chao Liu.

**Data curation:** Jing Bai, Lanchun Liu.

**Formal analysis:** Jing Bai, Jialiang Gao.

**Funding acquisition:** Jie Wang.

**Methodology:** Chao Liu, Lanchun Liu.

**Project administration:** Jie Wang.

**Resources:** Jing Bai.

**Software:** Lanchun Liu, Jialiang Gao.

**Writing – original draft:** Chao Liu, Lanchun Liu.

**Writing – review & editing:** Chao Liu, Jing Bai.
